# Unexpected Spontaneous Regression of Extensively Diffused Hepatocellular Carcinoma

**DOI:** 10.7759/cureus.79366

**Published:** 2025-02-20

**Authors:** Taiyo Hirata, Shinya Endo, Naofumi Shirane, Shinya Kawaguchi, Kazuya Ohno

**Affiliations:** 1 Gastroenterology, Shizuoka General Hospital, Shizuoka, JPN

**Keywords:** chronic hepatitis c, hepatocellular carcinoma, spontaneous regression, systemic inflammatory response, tumor hypoxia

## Abstract

Spontaneous regression (SR) of malignant tumors is defined as the partial or complete reduction of a tumor without medical intervention. This unusual phenomenon has been reported in various types of malignancies. We present a rare case of a 73-year-old woman with untreated chronic hepatitis C who was diagnosed with extensively diffused hepatocellular carcinoma (HCC) accompanied by a tumor thrombus in the left branch of the portal vein. Remarkably, extensive tumor regression occurred under palliative care. This case highlights the potential mechanisms responsible for SR of HCC, including tumor hypoxia, immune activation, and systemic inflammatory responses, and emphasizes the need for further research to clarify these mechanisms.

## Introduction

Primary liver cancer is the sixth most common malignancy worldwide and the third leading cause of cancer-related mortality globally. Hepatocellular carcinoma (HCC) accounts for 75-85% of all primary liver cancers, constituting the majority of liver cancer diagnoses [1]. In 2019, approximately 747,000 new cases of HCC were reported worldwide, with 480,000 deaths attributed to the disease [2]. Despite advancements in treatment, the prognosis for HCC remains poor [3].

Hepatitis C virus (HCV) infection is a well-established risk factor for the development of HCC. Chronic HCV infection induces persistent hepatic inflammation and fibrosis, eventually leading to cirrhosis and HCC. Furthermore, it is known that untreated HCV-infected patients have an increased risk of developing HCC even in the absence of cirrhosis [4].

Spontaneous regression (SR) of malignant tumors is characterized by the partial or complete reduction of a tumor without medical intervention. The overall frequency of SR across all malignancies is estimated to be one per 60,000-100,000 cases [5]. The mechanisms underlying SR in HCC are proposed to involve tumor hypoxia, immune activation, and systemic inflammatory responses [6]; however, the precise mechanisms remain uncertain. In this report, we describe a rare case of SR in a patient with diffuse HCC predominantly involving the left hepatic lobe, thus offering further insights into the potential causes of SR.

## Case presentation

**Figure 1:**
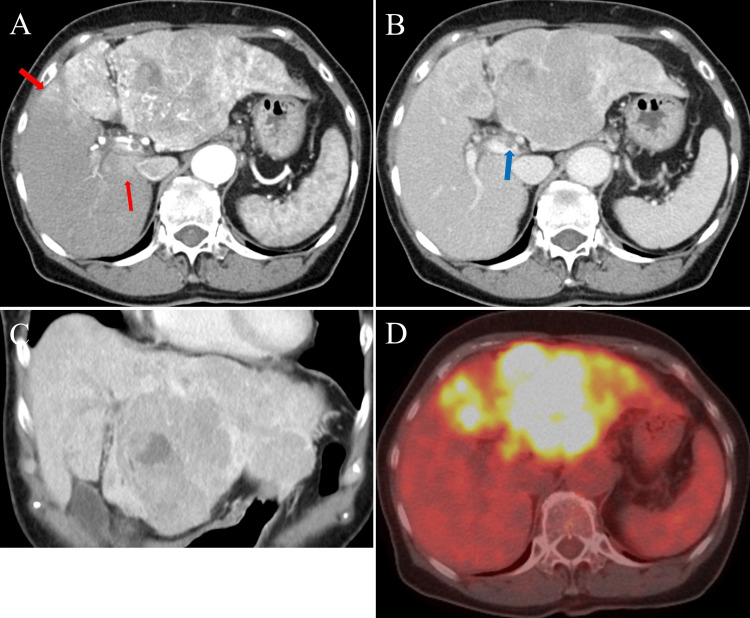
CT images at initial diagnosis A-C: CT images showed an irregularly cirrhotic liver with a large, 150-mm diffuse hepatocellular carcinoma (HCC) replacing the left lobe. The tumor appeared hyperattenuated in the arterial phase and hypodense in the portal venous phase. Multiple smaller HCC lesions were observed in the right lobe (shown by the red arrow). A tumor thrombus was visible in the left branch of the main portal vein (shown by the blue arrow). No lymph node involvement or distant metastases were identified. D: 18F-Fluorodeoxyglucose positron emission tomography/CT showing metabolic activity in the tumor-affected regions identified on CT.

The patient was a 73-year-old Japanese woman with untreated chronic hepatitis C who presented to our hospital with complaints of abdominal distension and a 5 kg weight loss over the course of one month. She had no significant past medical history apart from hepatitis C, with a smoking history of 37.5 pack-years and occasional alcohol consumption. At the presentation, she was afebrile and her vital signs were stable. Physical examination revealed abdominal distension and mild tenderness in the upper abdomen, but there was no evidence of asterixis or lower extremity edema. Initial multi-detector computed tomography (MDCT) demonstrated an irregular, cirrhotic liver with a large, 150-mm diffuse HCC replacing the left lobe. The lesion appeared hyperattenuated in the arterial phase and hypodense in the portal venous phase. Multiple smaller HCC lesions were observed in the right lobe, and a tumor thrombus was identified in the left branch of the main portal vein (Figures 1A-1C). 18F-fluorodeoxyglucose-positron emission tomography/computed tomography (18F-FDG PET/CT) revealed metabolic activity in these areas (Figure 1D). There was no evidence of lymph node or distant metastasis.

Laboratory findings showed total bilirubin (T.Bil) 1.3 mg/dL, aspartate aminotransferase (AST) 79 U/L, alanine aminotransferase (ALT) 67 U/L, albumin (Alb) 3.5 g/dL, and prothrombin time-international normalized ratio (PT-INR) 1.2. She was positive for hepatitis C virus (HCV) antibodies, with an HCV RNA level of 5.8 log IU/mL. Tumor markers were markedly elevated, including α-fetoprotein (AFP) 40,948 ng/mL and protein induced by vitamin K absence or antagonist-II (PIVKA-II) 18,954 mAU/mL. Her platelet count was reduced (122,000/μL), but her other organ functions were preserved. Her hepatic reserve was classified as Child-Pugh grade B (score: 8), and she was staged as Barcelona Clinic Liver Cancer (BCLC) stage C [7]. Despite being eligible for systemic chemotherapy or other aggressive treatments, the patient opted for palliative care with observation. She was not prescribed any medications except for analgesics, and she ceased her 37.5-pack-year smoking habit immediately following her diagnosis of HCC.

**Figure 2:**
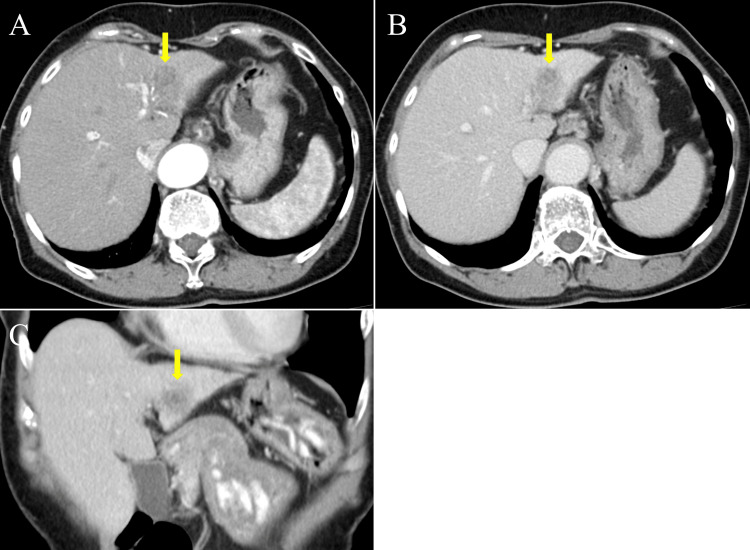
CT images seven months after HCC diagnosis A-C: Follow-up CT images showed marked regression of the previously hypervascular hepatocellular carcinoma (HCC) with washout, which had occupied the entire left lobe. Only a residual low-attenuation area, suggesting a possible remaining lesion, was observed in the lateral segment of the left lobe (shown by the yellow arrow). The tumor thrombus in the left branch of the main portal vein had disappeared.

Follow-up MDCT seven months after the initial diagnosis revealed marked regression of the hypervascular HCC, which had previously occupied the entire left hepatic lobe, with only a residual low-attenuation area suggesting a possible remaining lesion observed in the lateral segment of the left lobe. The tumor thrombus in the left branch of the main portal vein had disappeared (Figures 2A-2C).

Her tumor markers were notably reduced, with AFP 39 ng/mL and PIVKA-II 34 mAU/mL. Blood tests indicated improved liver function, including T.Bil 0.8 mg/dL, AST 97 U/L, ALT 114 U/L, Alb 3.7 g/dL, and PT-INR 1.14. The hepatic reserve improved to Child-Pugh grade A (score: 5). 

**Figure 3:**
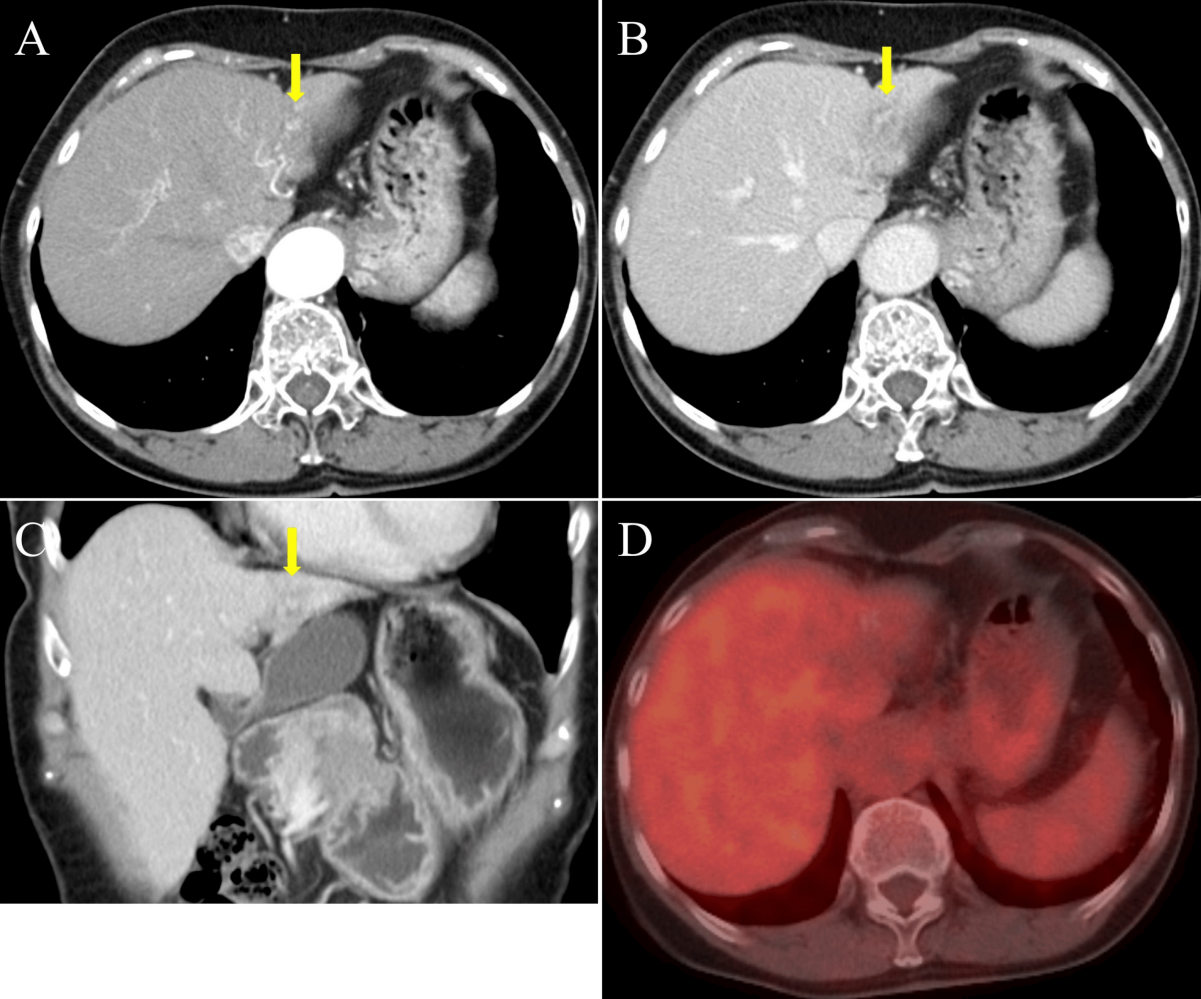
CT images 18 months after HCC diagnosis A-C: Follow-up CT images showed further regression of the left lobe, with a decrease in the size of the residual low-attenuation area in the lateral segment (shown by the yellow arrow). D: 18F-Fluorodeoxyglucose (FDG) positron emission tomography/CT showed regression of the previously observed hypermetabolic tumors, with no pathological FDG uptake in the liver. HCC: Hepatocellular carcinoma

Follow-up MDCT 18 months after the initial diagnosis demonstrated further shrinkage of the left lobe, with additional reduction of the residual low-attenuation area in the lateral segment (Figure 3A-3C). 18F-FDG PET/CT showed that the previously observed hypermetabolic HCC lesions had regressed, and there was no pathological FDG uptake in the liver. These imaging findings suggested that all previously identified HCC lesions had nearly disappeared (Figure 3D).

Her tumor markers remained stable, with AFP 41 ng/mL and PIVKA-II 27 mAU/mL, with no signs of an upward trend. The patient subsequently expressed an interest in hepatitis C treatment and underwent antiviral therapy with sofosbuvir/velpatasvir, achieving a sustained virologic response. Follow-up imaging studies indicated that the residual low-attenuation area in the lateral segment of the left lobe remained unchanged, suggesting post-regression changes of HCC rather than active disease progression. Imaging studies conducted 54 months after the initial diagnosis continued to show no evidence of HCC recurrence. Her tumor markers also remained within normal ranges, maintaining an unexpected clinical improvement (Table 1).

**Table 1:** Laboratory and Tumor Marker Trends T.Bil: Total bilirubin; AST: Aspartate aminotransferase; ALT: Alanine aminotransferase; Alb: albumin; PT-INR: Prothrombin time-international normalized ratio; PIVKA-II: Vitamin K absence or antagonist-II.

Laboratory Parameter	Normal Range	Initial Diagnosis	Seven Months Follow-up	18 Months Follow-up	54 Months Follow-up
T.Bil (mg/dL)	0.4 - 1.5	1.3	0.8	0.9	0.8
AST (U/L)	13 - 30	79	97	84	32
ALT (U/L)	7 - 23	67	114	116	34
Alb (g/dL)	4.1 - 5.1	3.5	3.7	3.9	4.4
PT-INR		1.20	1.14	1.09	1.08
AFP (ng/mL)	< 10	40948	39	41	7
PIVKA-II (mAU/mL)	< 40	18954	34	27	35

## Discussion

SR of malignant tumors, defined as the partial or complete reduction of malignancy without specific therapeutic intervention, was first described in 1956 [8]. SR was initially considered to be more common in certain cancers, such as renal cell carcinoma, malignant melanoma, neuroblastoma, and malignant lymphoma [9]; however, an increasing number of cases of SR of HCC have been documented since the first report of SR of HCC in 1972 [10]. A review by Chohan et al. [11] summarizing 106 cases of SR of HCC reported complete regression in 50.9% of cases. The average tumor size was 57 mm (range 15-130 mm), and the etiologies of HCC were predominantly hepatitis C (38.7%), hepatitis B (15.1%), and alcohol-related liver disease (11.3%), with cirrhosis observed in 32 cases (30.2%). The present case involved a diffuse HCC, measuring 150 mm and replacing the entire left hepatic lobe, in a patient with untreated chronic hepatitis C. Despite no aggressive treatment for the HCC, serial imaging with MDCT and 18F-FDG PET/CT, as well as tumor marker evaluations, demonstrated complete SR. This case is exceedingly rare compared with previous reports of SR of HCC because of the extensive and diffuse nature of the tumor, which highlights the uniqueness of this complete regression.

The mechanisms contributing to SR in HCC remain speculative, but several theories have been proposed. Tumor hypoxia is one possible mechanism, based primarily on vascular disruption, with portal vein thrombosis [12], rapid tumor growth [13], or large arteriovenous shunts [14] leading to a sudden reduction in hepatic blood flow and subsequent necrosis. This process may mimic the therapeutic effects of transarterial chemoembolization (TACE). Another proposed mechanism involves systemic inflammatory responses triggered by immune-mediated reactions. Factors such as smoking cessation and alcohol abstinence [15,16] or prolonged fever [17] can induce systemic inflammatory responses that could potentially lead to SR of HCC. Previous reports of HCC cases with SR have also documented elevated serum levels of interleukin-18 [18] and tumor necrosis factor-α [19], supporting the role of immune reactions in this phenomenon. In the present case, the tumor thrombus in the left branch of the main portal vein likely caused ischemia and subsequent necrosis of the tumor. Furthermore, the patient’s decision to quit smoking may have reduced oxidative stress and enhanced their natural immune responses against the tumor, contributing to SR. These findings suggest that both tumor hypoxia and immune activation may have played critical roles in the SR in the current case.

Regarding the prognosis of patients with SR of HCC, previous studies reported a median progression-free survival of 51 months and a median overall survival of 83 months in 66 patients who received no additional treatment after SR [11]. Conversely, however, a mini-review summarizing 24 cases found that five of 13 patients who did not receive any additional treatment experienced residual tumors or recurrence, compared with recurrence in only one of 11 patients who underwent additional treatments such as surgery, TACE, or radiotherapy [20]. The need for additional treatments after SR of HCC thus remains a topic for debate, but additional treatments such as surgery, TACE, or radiotherapy may be viable options for patients with an acceptable performance status and liver function and who express a desire for further intervention. Following complete regression of the HCC, the current patient received antiviral therapy with sofosbuvir/velpatasvir as part of the treatment for hepatitis C, but opted for observation without additional interventions for HCC. Remarkably, there was no evidence of recurrence 54 months after the initial diagnosis, reflecting a favorable clinical outcome.

## Conclusions

Spontaneous regression of HCC is a rare and poorly understood phenomenon. This case suggests that tumor hypoxia and immune-mediated systemic inflammation may contribute synergistically to achieve complete regression. Further reports could improve our understanding and potentially inform novel therapeutic strategies for cases where standard treatments are not viable.
